# Psychological distress and its influencing factors among psychiatric nurses in China: A cross-sectional study

**DOI:** 10.3389/fpsyt.2022.948786

**Published:** 2022-08-17

**Authors:** Juan Wang, Zhongren Zheng, Yingxue Tang, Rui Zhang, Qinghua Lu, Bin Wang, Qihua Sun

**Affiliations:** ^1^School of Public Health, Weifang Medical University, Weifang, China; ^2^School of Clinical Medicine, Jining Medical University, Jining, China; ^3^School of Nursing, Weifang Medical University, Weifang, China; ^4^School of Nursing, Shandong First Medical University and Shandong Academy of Medical Sciences, Jinan, China; ^5^Department of Infection Management, Shandong Mental Health Center, Jinan, China; ^6^Psychology Department, The Affiliated Provincial Hospital of Shandong First Medical University (Shandong Provincial Hospital), Jinan, China; ^7^Shandong Mental Health Center, Jinan, China

**Keywords:** psychiatric nurse, psychological distress, sleep quality, coping styles, China

## Abstract

**Background:**

Psychiatric nurses often face abuse, attacks, escape, suicides, and other situations related to the care of patients with mental disorders, which are more likely to induce psychological distress.

**Aims:**

This study aimed to examine the relationship between coping styles and psychological distress among Chinese psychiatric nurses in Shandong and the significance of sleep quality as a mediating factor.

**Methods:**

A total of 812 psychiatric nurses in Shandong, China, were investigated using the Psychological Distress Scale (K10), Simplified Coping Style Questionnaire (SCSQ), Pittsburgh Sleep Quality Index (PSQI) and self-compiled general information questionnaire.

**Results:**

Psychological distress was detected in 571 psychiatric nurses (70.3%). The psychological distress of psychiatric nurses was significantly different with respect to professional title (χ^2^ = 10.627, *P* < 0.05) and shift work (χ^2^ = 9.120, *P* < 0.01). Psychological distress positively correlated with negative coping style (*r* = 0.266, *P* < 0.01) and sleep quality (PSQIT) (*r* = 0.532, *P* < 0.01). A significant positive correlation was found between psychological distress and all dimensions of sleep quality (*r* = 0.158–0.456, *P* < 0.05). Professional title, positive coping style, negative coping style, sleep quality (PSQIT), subjective sleep quality, sleep disorder and daytime dysfunction predicted psychological distress in psychiatric nurses (*R*^2^ = 0.363, *F* = 65.343, *P* < 0.01). The relationship between negative coping style and psychological distress was partially mediated by sleep quality, with the mediating effect accounting for 37.97% of the total effect.

**Conclusions:**

Psychiatric nurses have a high rate of psychological distress, which is closely related to coping styles, and sleep quality has a certain regulatory effect.

## Introduction

Psychological distress refers to a non-specific negative psychological state whereby individuals have a perceived inability to cope with stress (1, 2). Psychological distress presents with mild-to-moderate symptoms of depression and anxiety, which can lead to a more severe mental illness when undetected. A study reported that approximately 543 million people in 2016 experienced psychological distress because of anxiety and depression (3). With the increase in life expectancy and occupational stress worldwide, the prevalence of mental illness and psychological problems has increased. Studies have also found a higher frequency of mental issues in health care workers than in the common populace, and they are more prone to work stress and depression (4). Nurses face various occupational pressures and considerable psychological troubles. For example, witnessing the suffering and death of patients, medical errors and adverse events, complex interpersonal relationships, and occupational pressures such as promotion and examination; thus, nurses are prone to psychological distress (5). More than 70% of nurses experience anxiety, depression, tension, and dejection in their personal emotional life and work (6). Psychiatric nurses who took care of patients with mental disorders often face abuse, assaults, beatings, suicide, escape, and other situations; thus, nurses and patients with mental disorders should establish more complex interpersonal relationships. However, psychiatric nurses generally bear the greater psychological pressure and severe psychological distress (7). Thus, far, only a few large-sample studies have focused on psychological distress among psychiatric nurses.

Psychological distress is influenced by several factors, including mental growth (8), social support (9), symptom burden (10), unmet needs of the patients (11), sleep disorder (12, 13), and others. Coping styles have been proved to be a reliable psychological and behavioral technique for dealing with external and internal obstacles positively or negatively, which is closely related to psychological distress (14, 15). A positive coping style will more often than not be related to addressing issues directly and rationally, whereas a negative coping style is inclined to figure out problems by ignoring, avoiding, and denying (16). Roesch et al. (17) showed that an inclination to embrace a positive coping style valuably affects physical and psychological well-ness, whereas a negative coping style antagonistically affects physical and emotional well-being, resulting in psychological distress (18). This presents a huge connection between coping styles and psychological distress, that is, the degree of psychological distress is lower when a positive coping style is adopted, whereas the level of psychological distress is higher when a negative coping style is adopted.

Sleep is an integral part of health, growth, and survival (19). Sleep disorder refers to the abnormal quantity and quality of sleep, including reduced or excessive sleep and abnormal sleep-related behaviors, which influence personal satisfaction and physical and psychological well-ness (20). Insomnia is the most common sleep disorder among the general population, and 10−30% of the world's population are enduring insomnia (21). Insomnia is more common among Chinese nurses, especially psychiatric nurses (22). Kalmbach et al. found that participants diagnosed with insomnia had an increased risk of developing physical and mental disorders, including chronic pain, back pain, hypertension, diabetes, stroke, anxiety, and depression, compared with healthy participants (23). Moreover, studies have found that both poor sleep quality and insomnia may increase the risk of developing mental illness (24). People with poor sleep quality have a higher degree of psychological distress (25). Sleep disorders among nurses on shifting work schedules are caused by the dys-synchronization of the endogenous physiological system of the circadian rhythm. Fatigue and sleepiness worsened because of the decreased amount of sleep, leading to general physical and mental health problems (26). Moreover, an imbalance between the sleep-wake cycle and the endogenous circadian system aggravates various hormonal and metabolic processes that may adversely influence physical and emotional well-being; compared with day workers, shift workers are more likely to develop chronic illnesses such as cardiovascular, metabolic and psychological disorders (27). A link between sleep quality and psychological distress was reported, that is, insomnia and short sleep duration can decrease mood and augment anxiety, increasing psychological distress (28). The relationship between sleep quality and psychological distress is ascribed to the activation of the rapid eye movement (REM) sleep mechanism, as people with REM sleep behavior have a significantly increased risk of anxiety and depression (29). At present, only a few studies have examined the connection between sleep quality and psychological distress in the field of psychiatric nursing; thus, it is worthy of further investigation.

Despite studies on the effects of coping styles and sleep quality on psychological distress (30, 31), the underlying regulatory mechanisms are not well understood. These studies have shown that negative coping style not only directly affects psychological distress but also has mediating variables, such as exercise habits and experiential avoidance (32, 33). Thus, recent research presents that other underlying mediators or mechanisms are at play between negative coping style and psychological distress. With this, we hypothesized that sleep quality mediates negative coping style and psychological distress.

This study aimed to explore the relationship between sleep quality, coping styles, and psychological distress among psychiatric nurses. This study further explored whether sleep quality plays a mediating role between negative coping and psychological distress to provide theoretical support for nursing management in the psychiatric department and thereby improve the mental health of psychiatric nurses.

## Materials and methods

### Participants

**Figure 1:**
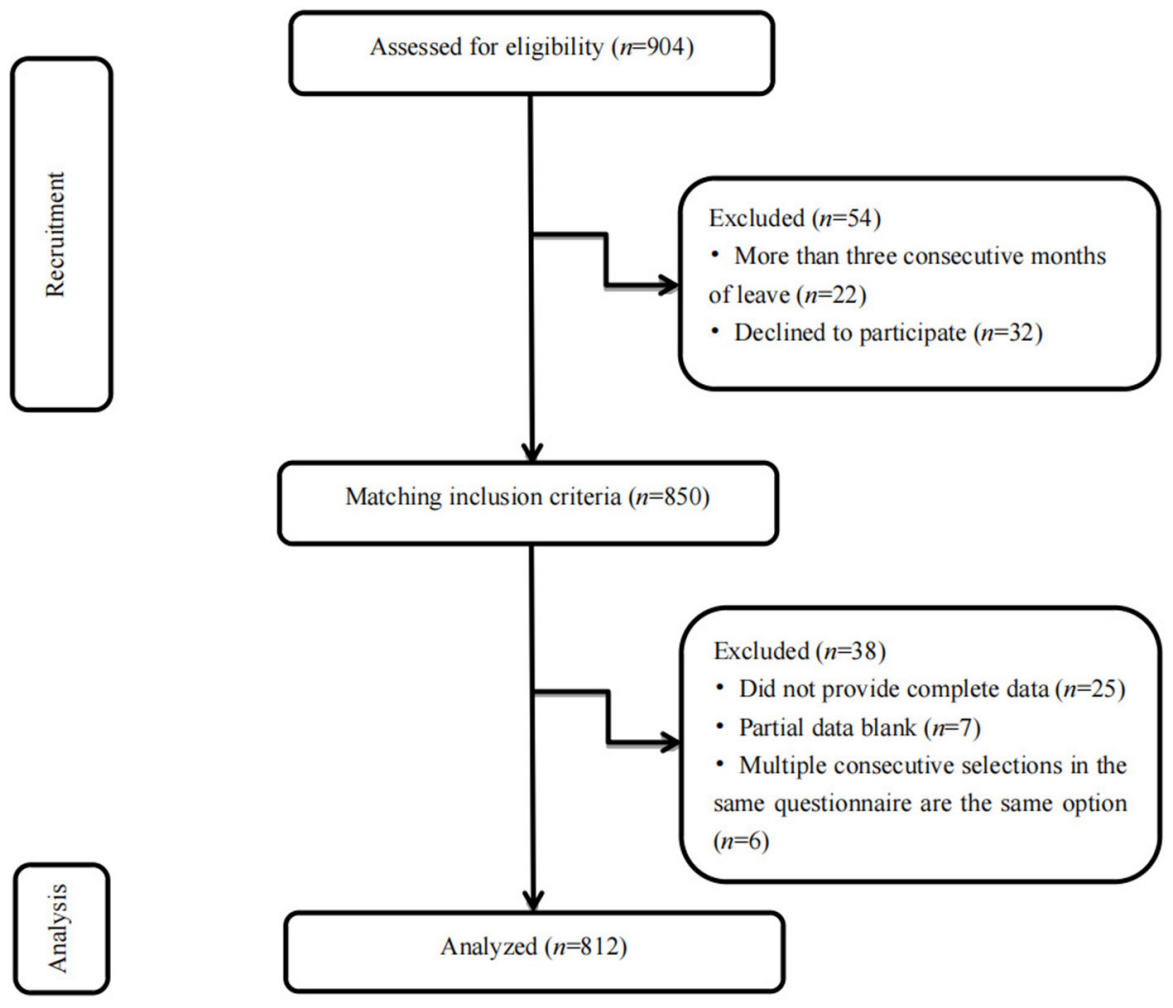
Flow diagram of subjects recruitment and study selection (*n* = 812, Shandong, China).

This was a cross-sectional study with stratified sampling performed between November to December 2020. According to the geographical characteristics of Shandong province, 17 prefecture-level cities were divided into six following areas: northwest, southwest, southern, northern, and central Shandong province and the Shandong Peninsula. A tertiary psychiatric hospital was randomly selected from the counties and cities in the six geographical areas using a random number table. All clinical nurses in the hospital were the study subjects. A total of 904 clinical nurses were randomly enrolled. The inclusion criteria were as follows: (1) 18–60 years with no sex or marital status restrictions; (2) working duration of at least 1 year; (3) national nurse practice registration qualification and practice registration in the unit; (4) clinical ward nurses; and (5) those who were on duty and volunteered for the investigation. The exclusion criteria were as follows: (1) those younger than 18 years or older than 60 years; (2) working duration of < 1 year; (3) non-official staff of the unit including advanced, practicing, and visiting nurses; (4) those on leave for more than three consecutive months; and (5) those who refused to participate in the survey (Figure 1).

### Ethical issues

In this study, the researcher firstly conducted online training for the participants to introduce basic information and fill in the requirements of the questionnaire in detail. The questionnaire strictly followed the principle of confidentiality, and all participants provided written informed consent before the study. This study was approved by the Ethics Committee of Shandong Mental Health Center. During the review process, privacy was completely safeguarded. The study protocol followed the moral norms of the 1964 Declaration of Helsinki.

### Measures

#### General information

Age, sex, marital status, educational level, professional title, income, work shift and working duration were defined in the general information questionnaire.

#### Psychological distress

The 10-item Kessler Psychological Distress Scale (K10), compiled by Kessler et al. (34) and revised by Zhou et al. (35) (Chinese version of the revised scale comprises 10 items and evaluates an individual's level of psychological distress on a scale of 1 to 5) was used to evaluate the psychological distress. A score of ≥16 on this scale indicates the presence of psychological distress; 10–15 indicates almost no psychological distress; 16–21 indicates mild psychological distress; 22–29 indicates moderate psychological distress, and 30–50 indicates severe psychological distress. Thus, the higher the score, the more severe the psychological distress. In this study, Cronbach's α of the scale was 0.930.

#### Simplified coping style questionnaire (SCSQ)

The SCSQ was developed by Xie (36) and included 20 items for the positive and negative coping styles. The Chinese version of the questionnaire was used to evaluate and reflect the characteristics of different coping styles among populations. To measure the characteristics of a positive coping style, 12 items were assessed. For instance, items included: “seeking advice from relatives, friends, or classmates” and “seeking interests and being active in recreational and sports activities.” The measure for the negative coping style dimension comprised 8 items. Examples included: “trying to take a break or vacation to put the problem (worry) behind you for a while” and “trying to forget the whole thing.” Cronbach's α coefficient of 12 items of the positive coping style was 0.890, whereas that of the eight items of the negative coping style was 0.780. The retest reliability of the scale was 0.890. Cronbach's α of the negative coping style was 0.809 in this study.

#### Pittsburgh sleep quality index (PSQI)

The PSQI, compiled by Buysse et al. (37) has been tested for reliability and validity in China by Liu et al. (38) and is utilized to assess the sleep quality of participants within the last month. The Chinese edition of the PSQI was viewed as a substantial measure of sleep quality. The Chinese version of the questionnaire, suitable for domestic research, was used in this study to assess each dimension. This index included seven factors, namely, subjective sleep quality, sleep latency, sleep persistence, habitual sleep efficiency, sleep disorders, use of hypnotic drugs and day-time dysfunction. Each factor was scored 0–3, and the cumulative score of each component represents the PSQI total score, ranging from 0 to 21 points. Patients were characterized as individuals who scored >7 points, and those who scored ≤ 7 points were non-patients. Thus, the higher a person's score, the worse the sleep quality. Cronbach's α was 0.839.

### Statistical analysis

IBM SPSS Statistics for Windows, version 25.0 (IBM Armonk, NY, USA) was used for data entry and statistical analysis, and *P* < *0.05* was considered significant. The univariate analysis was performed using the *t*-test and chi-square test to evaluate the relationship between sample characteristics and variables. Continuous variables were expressed as mean and standard deviation, whereas categorical variables were expressed as frequency and percentage. The correlations among the negative coping style, sleep quality and psychological distress were analyzed using Pearson correlation, multiple stepwise regression analysis, structural equation modeling, R 4.1.2, and non-parametric percentile bootstrap test.

## Results

### Sample characteristics

Of the total 904 questionnaires sent out, 850 were collected, and 812 were valid, of which the effectiveness rate was 89.8%. The average age of the 812 psychiatric nurses was 32.69 ± 8.07 (range, 18–58) years. Of the respondents, 208 were male (25.6%) and 604 were female (74.4%). Regarding marital status, 630 were married (77.6%) and 182 were not married (22.4%). A total of 471 participants (58.0%) held a bachelor's degree or higher, and 341 (42.0%) had a college degree or lower grade levels. Of the 812 respondents, 283 were nurses (34.9%), 294 (36.2%) were junior nurses, 193 (23.8%) were senior nurses, and 42 (5.2%) were associate superintendent nurses. Regarding the income, 243 earned < 3000 yuan (29.9%), 384 earned 3000–5000 yuan (47.3%) and 185 earned > 5000 yuan (22.8%). Furthermore, 130 participants were working day shifts (16.0%), whereas 682 had a three-shift rotation (84.0%). The working duration ranges from 1 to 40 years, with an average working duration of 6 years (*P*25 = 3 years, *P*75 = 15 years).

### Comparison of participant characteristics and K10 scores

The suffer group was defined as K10 ≥16 points, and K10 <16 as the non-suffer group. Moreover, 29.7% of the respondents had K10 scores <16 points, 571 (70.3%) scored ≥16, and 24.96 ± 6.85 was the average K10 score. Regarding scores of psychological distress, a significant difference was found in the professional title (χ*2* = 10.627, *P* < 0.05) and work shift (χ*2* = 9.120, *P* < 0.01), but age, sex, marital status, education level, income and working duration did not show any significant differences (Table 1).

**Table 1:** Sociodemographic characteristics and comparisons of the scores on K10 (*n* = 812, Shandong, China).

**Variables**		***N***	**K10≥16**	**Prevalence (%)**	***χ2***	***P***
Age	<30 years	379	259	68.34	1.517	0.468
	30–39 years	254	185	72.83		
	≥40 years	179	127	70.95		
Sex	Male	208	140	67.31	1.216	0.270
	Female	604	431	71.36		
Marital status	Married	630	446	70.79	0.302	0.583
	Single	182	125	68.68		
Educational level	College degree or below	341	229	67.16	2.821	0.093
	Bachelor's degree or above	471	342	72.61		
Professional title	Nurse	283	185	65.37	10.627	0.014
	Junior nurse	294	209	71.09		
	Senior nurse	193	151	78.24		
	Associate superintendent nurse	42	26	61.90		
Income	≤3000 yuan	243	160	65.84	3.595	0.166
	3000–5000 yuan	384	280	72.92		
	>5000 yuan	185	131	70.81		
Work shift	Day shift only	130	77	59.23	9.120	0.003
	Three-shift rotation	682	494	72.43		
Working duration	<3 years	158	98	62.03	7.041	0.071
	3–5 years	204	149	73.04		
	6–10 years	195	137	70.26		
	>10 years	255	187	73.33		

“Three-shift rotation” is a fixed rotating shift schedule, which consists of an 8-h day shift, an 8-h swing shift and an 8-h night shift.

### Relationship between PSQI, SCSQ and K10 among psychiatric nurses

Compared with non-sufferers (K10 < 16), the average positive coping style score of sufferers was 22.69 ± 6.02 (range, 0–36) (*t* = 4.029, *P* < 0.01), and the average negative coping style score of sufferers was 10.38 ± 4.75 (range, 0–24) (*t* = −5.865, *P* < 0.01). The average sleep quality (PSQIT) score of sufferers was 7.31 ± 3.69 (range, 0–19) (*t* = −13.941, *P* < *0.01*), whereas the average subjective sleep quality score of the sufferers was 1.33 ± 0.84 (range, 0–3) *(t* = −12.015, *P* < 0.01). The average sleep latency score of the sufferers was 1.47 ± 0.89 (range, 0–3) *(t* = −7.920, *P* < 0.01), whereas that of the sufferers was 1.30 ± 0.99 (range, 0–3) *(t* = −7.853, *P* < *0.01*). The average habitual sleep efficiency score and average sleep disorder score of the sufferers were 0.55 ± 0.88 (range, 0–3) (*t* = −3.964, *P* < *0.01*) and 1.26 ± 0.67 (range, 0–3) (*t* = −9.790, *P* < *0.01*), respectively. The average hypnotic drug use score and average daytime dysfunction score of the sufferers were 0.22 ± 0.64 (range, 0–3) (*t* = −5,465, *P* < *0.01*) and 1.16 ± 0.92 (range, 0–3) (*t* = −10.810, *P* < *0.01*), respectively (Table 2).

**Table 2:** Analysis of different groups of psychological distress among psychiatric nurses (*n* = 812, Shandong, China).

**Factor**	**Sufferers K10 ≥ 16**	**Non-sufferers K10 < 16**	***t***	***p***
	**Mean ±SD**	**Mean ±SD**		
Positive coping style	22.69 ± 6.02	24.58 ± 6.31	4.029	<0.01
Negative coping style	10.38 ± 4.75	8.37 ± 4.34	−5.865	<0.01
Sleep quality (PSQIT)	7.31 ± 3.69	4.22 ± 2.47	−13.941	<0.01
Subjective sleep quality	1.33 ± 0.84	0.69 ± 0.62	−12.015	<0.01
Sleep latency	1.47 ± 0.89	0.97 ± 0.81	−7.920	<0.01
Sleep persistence	1.30 ± 0.99	0.80 ± 0.73	−7.853	<0.01
Habitual sleep efficiency	0.55 ± 0.88	0.33 ± 0.65	−3.964	<0.01
Sleep disorder	1.26 ± 0.67	0.84 ± 0.52	−9.790	<0.01
Use hypnotic drugs	0.22 ± 0.64	0.05 ± 0.24	−5.465	<0.01
Daytime dysfunction	1.16 ± 0.92	0.54 ± 0.68	−10.810	<0.01

PSQIT-The total score of the Pittsburgh sleep quality index.

**Figure 2:**
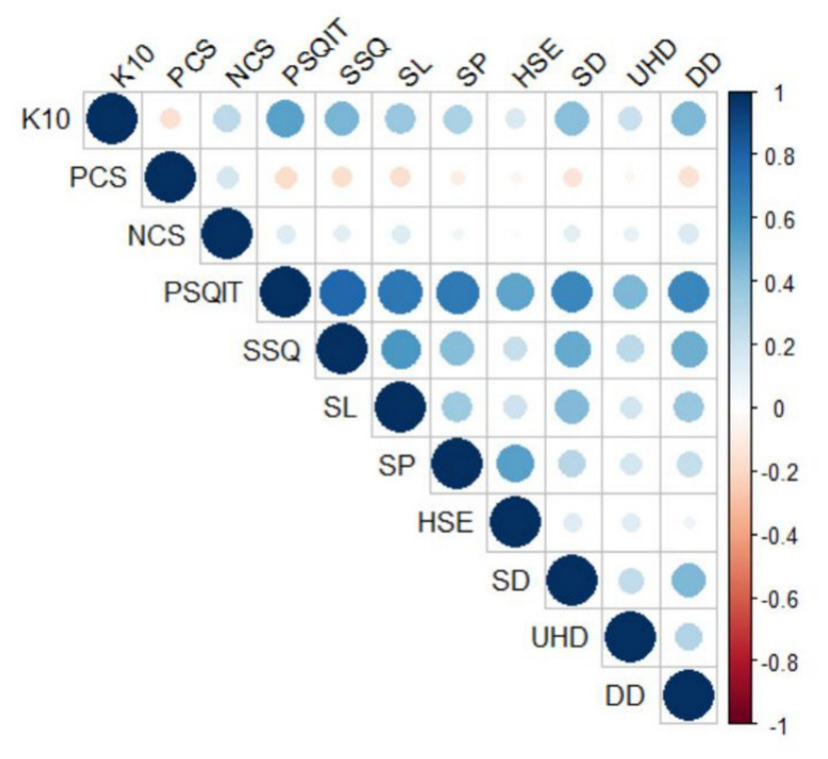
Heat map of the relationship between sleep quality and its various dimensions, coping styles and psychological distress among psychiatric nurses (*n* = 812, Shandong, China).

Psychological distress was positively correlated with the negative coping style (*r* = 0.266, *P* < 0.01), sleep quality (PSQIT) (*r* = 0.532, *P* < 0.01), and all dimensions of sleep quality (*r* = 0.158–0.456, *P* < 0.05) (Table 3). To more clearly describe the correlation between the indicators, we plot the correlation heat map using R 4.1.2, as displayed in Figure 2.

**Table 3:** Relationship between sleep quality and its various dimensions, coping styles and psychological distress among psychiatric nurses (*n* = 812, Shandong, China).

	**Psychological** **distress**	**Positive** **coping** **style**	**Negative** **coping** **style**	**Sleep** **quality** **(PSQIT)**	**Subjective** **sleep** **quality**	**Sleep** **latency**	**Sleep** **persistence**	**Habitual** **sleep** **efficiency**	**Sleep** **disorder**	**Use** **hypnotic** **drugs**	**Daytime** **dysfunction**
Psychological distress	1										
Positive coping style	−0.155^**^	1									
Negative coping style	0.266^**^	0.174^**^	1								
Sleep quality (PSQIT)	0.532^**^	−0.177^**^	0.137^**^	1							
Subjective sleep quality	0.456^**^	−0.164^**^	0.118^**^	0.782^**^	1						
Sleep latency	0.371^**^	−0.163^**^	0.133^**^	0.715^**^	0.575^**^	1					
Sleep persistence	0.318^**^	−0.083^*^	0.053	0.700^**^	0.426^**^	0.359^**^	1				
Habitual sleep efficiency	0.158^**^	−0.05	−0.027	0.524^**^	0.221^**^	0.202^**^	0.541^**^	1			
Sleep disorder	0.420^**^	−0.133^**^	0.111^**^	0.649^**^	0.502^**^	0.434^**^	0.277^**^	0.126^**^	1		
Use hypnotic drugs	0.212^**^	−0.044	0.090^*^	0.449^**^	0.265^**^	0.187^**^	0.175^**^	0.128^**^	0.240^**^	1	
Daytime dysfunction	0.447^**^	−0.146^**^	0.147^**^	0.650^**^	0.490^**^	0.374^**^	0.231^**^	0.061	0.449^**^	0.290^**^	1

PSQIT-The total score of the Pittsburgh sleep quality index;

** P < 0.01,

* P < 0.05.

### Predictors of psychological distress among psychiatric nurses

A multiple stepwise regression analysis was employed to analyse the predicting factors of psychological distress among psychiatric nurses (Table 4). The dependent variable was psychological distress, and the independent variables were professional title, shift work, positive coping style, negative coping style, sleep quality (PSQIT), subjective sleep quality, sleep latency, sleep persistence, habitual sleep efficiency, sleep disorder, use of hypnotic drugs and day-time dysfunction. Professional title (*β* = *0.084, P* < 0.01), positive coping style (*β* = −0.105, *P* < 0.01), negative coping style (*β* = 0.204, *P* < 0.01), sleep quality (PSQIT) (*β* = 0.235, *P* < 0.01), subjective sleep quality (*β* = 0.101, *P* < 0.05), sleep disorder (*β* = 0.119, *P* < 0.01) and daytime dysfunction (*β* = 0.142, *P* < 0.01) were factors associated with psychological distress (*R*^2^ = 0.363, *F* = 65.343, *P* < 0.01).

**Table 4:** Predictors factors of psychological distress in psychiatric nurses (*n* = 812, Shandong, China).

**Variable**	***B***	***SE***	**β**	***t***	***P***	**Tolerance**	**VIF**
(Constant)	12.152	1.171	–	10.382	<0.01	–	-
Professional title	0.768	0.259	0.084	2.964	<0.01	0.979	1.021
Positive coping style	−0.138	0.039	−0.105	−3.546	<0.01	0.911	1.098
Negative coping style	0.351	0.050	0.204	6.994	<0.01	0.932	1.073
Sleep quality (PSQIT)	0.522	0.126	0.235	4.129	<0.01	0.245	4.081
Subjective sleep quality	0.981	0.439	0.101	2.232	<0.05	0.387	2.584
Sleep disorder	1.471	0.460	0.119	3.201	<0.01	0.575	1.740
Daytime dysfunction	1.277	0.335	0.142	3.807	<0.01	0.568	1.760

R^2^ = 0.363, Adjusted R^2^ = 0.357, F = 65.343, P < 0.01.

### Sleep quality as a mediating factor between negative coping style and psychological distress

To test our hypothesis that sleep quality mediates the relationship between negative coping style and psychological distress, we performed a stratified regression analysis. The independent variable was the negative coping style (X), the mediating variable was sleep quality (M) and the dependent variable was psychological distress (Y). First, the regression equation coefficient c of negative coping on psychological distress demonstrated significance (*P* < 0.01), and the influence coefficient a of negative coping style on sleep quality also presented significance (*P* < 0.01). Finally, the influence coefficients c' and b of sleep quality and negative coping style on psychological distress were significant (*P* < 0.01). This finding suggests that sleep quality partially mediates the negative coping style and psychological distress (Table 5).

**Table 5:** Stratified regression models for sleep quality, negative coping style and psychological distress among psychiatric nurses (*n* = 812, Shandong, China).

**Analysis and model**	**Dependent variable**	**Independent variable**	***b***	***b'***	***R^2^***	**Adjusted R^2^**	***t***	***p***
Y = cX + e1	K10	NCS	0.458	0.266	0.266	0.070	7.868	<0.01
M = aX + e2	SQ	NCS	0.106	0.137	0.137	0.018	3.941	<0.01
Y = c'X + bM + e3	K10	NCS	0.339	0.197	0.567	0.319	6.743	<0.01
		SQ	1.121	0.505	0.567	0.319	17.255	<0.01

NCS, negative coping style; SQ, sleep quality (PSQIT).

**Figure 3:**
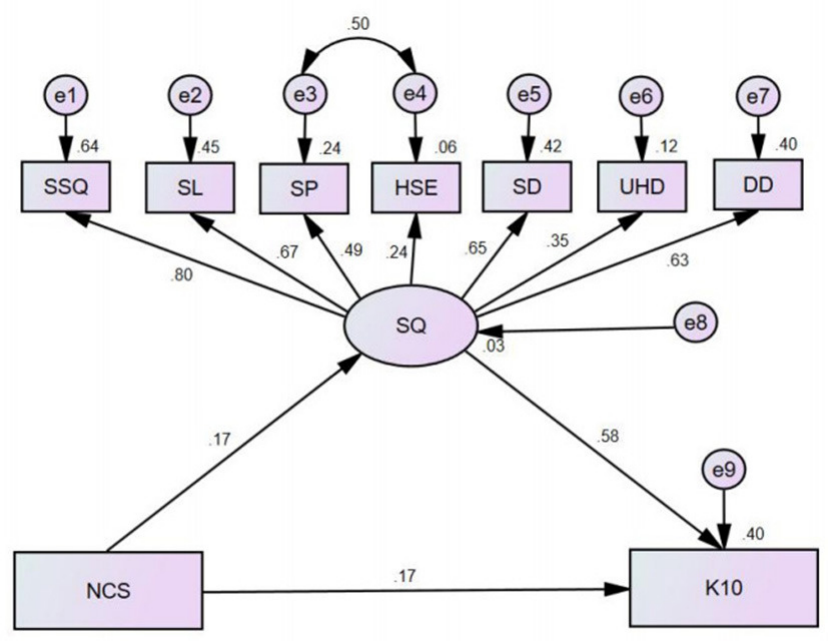
Standardized estimates of the relationship between negative coping style, sleep quality, and psychological distress (*n* = 812, Shandong, China).

Based on the results of the stratified regression analysis, we further constructed a structural equation model of the relationship between negative coping style, sleep quality and psychological distress (Figure 3). The original fit parameters of this model were as follows: χ^2^ = 296.972, root mean square error of approximation (RMSEA) = 0.113, normed fit index (NFI) = 0.838, comparative fit index (CFI) = 0.849, goodness of fit index (GFI) = 0.926, adjusted goodness of fit index (AGFI) = 0.871 and chi-square/degrees of freedom (GMIN/DF) = 11.422. These parameters demonstrated that the model was not appropriate. After adjusting the model, its fitness was good on the basis of the following parameters: χ^2^ = 79.171, RMSEA = 0.052, NFI = 0.957, CFI = 0.970, GFI = 0.978, AGFI = 0.961 and GMIN/DF = 3.167. The difference in each route structure was significant (*P* < 0.01). Negative coping style and sleep quality have direct, indirect and overall consequences on psychological distress, as shown in Table 6. According to the findings, a negative coping style had both direct and indirect effects on psychological distress, whereas sleep quality had only direct effects on psychological distress. Subjective sleep quality had the highest absolute value (0.80) among the seven dimensions of sleep quality, followed by sleep latency (0.67).

**Table 6:** Mediating effect of sleep quality between negative coping style and psychological distress among psychiatric nurses (*n* = 812, Shandong, China).

**Variables**	**Total effect**	**Direct effect**	**Indirect effect**
NCS scores	0.266	0.166	0.101
Sleep quality	0.578	0.578	0.000

NCS, negative coping style.

## Discussion

### Analysis of the status quo of psychological distress among psychiatric nurses

This study found that the incidence of psychological distress among psychiatric nurses was 70.3%. Moreover, 571 participants had a K10 score of ≥16, with an average score of 24.96 ± 6.85. The incidence of psychological distress observed in this study was lower than that reported by Liu et al. (39), which was 83.8%. The difference was possibly due to the inclusion of samples from six geographic regions in Shandong. Compared with patients admitted in provincial hospitals, patients with mental disorders treated in municipal hospitals have milder symptoms and the pressure on nurses is minimal. Moreover, compared with nurses in provincial hospitals, the number of nurses with higher positions, ongoing promotion and continuing education are small; thus, studies have only reported a low incidence of psychological distress among psychiatric nurses. In the present study, the occurrence of psychological distress among psychiatric nurses was lower than that of college students evaluated by Li et al. (40), which was 81.4%. The possible reason is that psychiatric nurses are becoming more stable professionally and have higher education and stable work than college students. On the contrary, college students are still in the process of developing professionally and going to school, and their families have very high expectations of them, resulting in a higher degree of psychological distress. Meanwhile, psychiatric nurses have been engaged in psychiatric psychology and mental health education for a long time and have self-regulation ability when experiencing psychological distress; thus, the incidence of psychological distress in psychiatric nurses is low.

Furthermore, a univariate study of sociodemographic data revealed that psychological distress was not influenced by age, sex, marital status, education, income, and working duration. However, it was strongly linked to professional title and shift work. This could be related to the fact that nurses bear multiple pressures from family, occupation and society simultaneously. In addition, professional nurses are exposed to constantly high levels of pressure, resulting in psychological distress (41). A study revealed that work pressure is emphatically connected with depression among nurses (42). Moreover, junior nurses have better physical fitness, high energy levels, adjustment ability and lower incidence of psychological distress. Nurse supervisors and those with higher positions have stable family, occupational, and social status, rich nursing experience, emergency response ability and lower incidence of psychological distress. Our study revealed that 72.43% of shift nurses had psychological distress, whereas 59.23% of day nurses have psychological distress and found a link between psychological distress and work shift. Studies have shown that shift work increases the risk of poor mental health and that shift employees have a 33% higher risk of experiencing poor mental state and depression than non-shift workers. Shift work can lead to changes in sleep patterns and mood changes such as depression, anxiety, irritability and nervousness (43). Studies have found that shift work in nursing disturbs the circadian rhythm, increases day-time sleepiness and reduces personal readiness, resulting in poor general, physical and mental health and the occurrence of sleep disorders, ultimately reducing work efficiency and affecting the quality of care (44). Psychiatric nurses are commonly at risk for the development of mental illness. Adverse events such as savage attacks, suicide and harm by the patients often occur at night; thus, psychiatric nurses are always in a high state of tension at work, leading to psychological stress.

### Correlation analysis of sleep quality, negative coping style and psychological distress among psychiatric nurses

This study showed that among psychiatric nurses, sufferers (K10≥16) and non-sufferers of psychological distress (K10 < 16) demonstrated significant differences in coping styles, the total score of sleep quality and its various dimensions. A negative coping style was positively associated with sleep quality and psychological distress. Negative attitudes and emotions will affect the development of physical and mental health. Moreover, a negative coping style is linked to more mental health issues, whereas a positive coping style is related to fewer mental health issues (45). The possible reason is the tendency to adopt negative coping strategies in the face of problems, which will increase the number of negative events. These individuals have a relatively negative view of the problems and choose to withdraw, disengage and refuse to communicate with others, with their emotions as the center, thus intensifying psychological pressure and leading to psychological distress. Therefore, when faced with negative events, nurses should adopt positive coping strategies, focus on the problem, choose humor and actively communicate with others.

Studies have shown that insomniacs often adopt negative and immature coping styles (46); excessive avoidance will affect sleep quality and generate more pressure, which will lead to psychological distress (47). Psychological distress among psychiatric nurses was positively connected with sleep quality and its various components. The poorer the sleep quality, the higher the degree of psychological distress; this corresponds with the findings of Kain and Caldwell-Andrews (48) and Roberts et al. (49). Thus, the poorer the sleep quality, the greater the interference with the normal pace of study, work and family life, ultimately increasing stress and psychological distress. Furthermore, individuals create negative feelings such as anxiety and depression that can disrupt the balance between central sympathetic and cholinergic activities, affect the regulation of sleep by the brain, prolong fast-wave sleep and then lead to sleep disorders. Sleep disorders are considered secondary symptoms of mental illness (50). Sleep is assumed as an indispensable part of mental well-being and is in a dynamic interaction with psychological distress. Therefore, nurses should gain relevant knowledge of sleep, understand the mechanism between sleep and mental well-being, reduce sleep problems and ultimately avoid events that induce psychological distress.

### Sleep quality as a mediator factor between negative coping style and psychological distress

The results of the stratified regression analysis and Amos model revealed that the sleep quality of psychiatric nurses partially mediated the connection between negative coping style and psychological distress. This indicated that negative coping can directly affect psychological distress and sleep quality can indirectly affect psychological distress, in which sleep quality plays a certain regulatory role.

According to a previous study, a delayed peak sleep-wake rhythm will cause a vicious cycle of circadian rhythm disorders and poor sleep, which will increase the incidence of anxiety and depression (51). Moreover, among patients with depression, some have increased plasma cortisol secretion, the altered circadian rhythm of cortisol secretion and spontaneous inhibition of cortisol secretion at night. The use of drugs such as dexamethasone will not inhibit cortisol production but leads to depression (52). Studies have found that improved sleep effectively reduced psychological stress and vice versa. A connection was found between sleep quality and psychological distress, and sleep deprivation was related to a series of negative health and psychosocial consequences, showing reciprocal or bidirectional relationships between the two (53). That is, if sleep desynchronisation occurs, a person will experience drowsiness, nocturnal insomnia, significantly reduced arousal threshold and easy wakefulness, followed by anxiety, depression, restlessness and irritability. Conversely, when a person is anxious, depressed, restless or disappointed, he/she is forced to fall asleep, which will aggravate the original sleep disorders.

Studies have found that sleep disorders, anxiety disorders, and depressive disorders were all related to the functional activity of serotonin, which is an important bridge between circadian rhythm disruption and depression. Furthermore, a bidirectional communication was found between serotonin and the circadian system, and sleep disorders, anxiety disorders and depression disorders were all related to the regulation of the circadian system. Numerous physiological cycles and behaviors, including the sleep-wake cycle, positive and negative aspects of sentiment, etc. are controlled by circadian rhythms (54). The disruption of the circadian rhythm environment had been shown to affect sentiment and increase the chance of mental illnesses (55). Compared with healthy controls, the circadian rhythm regulation mechanism has fewer effects on positive emotion, but it has strong effects on negative emotion (54). Another study showed that shift work is associated with bad moods and that shift workers have a higher chance of becoming depressed (56). Major life events, including divorce, death, shift work, childbirth and others affect daily lifestyle, which usually influences the circadian system; a disrupted circadian system may lead to sleep disorders, negative emotions and psychological distress (57). This suggested that the circadian rhythm regulation system played an important role in sleep disorders, coping with mood and psychological distress.

The findings revealed that coping styles are linked to psychological distress and that sleep quality had a regulatory effect. Coping styles, defined as a person's habitual inclination to solve difficulties or a common method for dealing with stressors, were found to be reasonably consistent. Psychological distress can be decreased to some extent by adopting steps to improve the sleep quality of psychiatric nurses. For the protection from perceived stress, good sleep quality is a valuable resource. Psychological distress and mental health can be alleviated by improving sleep quality (58). Nursing managers can improve the sleep quality of psychiatric nurses by providing reasonable work schedules and humanized care, adopting group intervention strategies such as mindfulness and encouraging them to use positive coping strategies to lessen psychological distress and enhance mental health.

### Limitations

This study has some limitations. First, although this study has enrolled samples from six geographical regions in Shandong Province, which has geographical and economic advantages, this study is not nationally representative. Second, given the cross-sectional study design, researchers may be more susceptible to recollection bias, making it hard to infer a causal link from the findings. Finally, this study used a self-reported questionnaire, which may affect the results. In future studies, we will further consider a national longitudinal analysis of sleep quality, coping styles, and psychological distress among psychiatric nurses.

## Conclusions

In summary,

1. Psychological distress was detected in psychiatric nurses at 70.3%. Significant associations were found with the professional title and work shift but age, sex, marital status, education level, income, and working duration showed no such trends.
2. Psychological distress correlated positively with the negative coping style, sleep quality, and all dimensions of sleep quality. Negative coping style and sleep quality were the predictors of psychological distress among psychiatric nurses.
3. Sleep quality partially mediated the relationship between negative coping style and psychological distress among psychiatric nurses.

## Implications

Following are the implications of the findings of this study:

1. We examined psychological distress' relationship with sleep quality and negative coping styles among nurses in six tertiary psychiatric hospitals in Shandong Province, China. Our findings indicated that psychological distress was prevalent among 70.3% of the psychiatric nurses. Zou et al. reported that 85.5% of the nurses in general hospitals suffer from psychological distress (5). Dong et al. found that the incidence of sleep disorders among nurses in general hospitals was 63.9% (41). Previous research has shown that the levels of anxiety and depression reduce significantly upon interventions that include structured relaxation; attention and explanatory therapy, and stress management and resilience training programs (59). Therefore, to better guide the clinical practice of nurses, studies on relevant interventions are needed to improve their mental health.
2. Sleep quality is the basic physiological need of the human body and is correlated with psychological distress. Nursing managers should be encouraged to pay attention to the sleep quality of nurses, and related treatments, including sleep relaxation therapy, mindful self-compassion training, kindness meditation therapy, and compassion focus therapy should be emphasized in clinical continuing education to improve the sleep quality of nurses, thus improving their psychological problems.
3. Coping styles are means by which individuals deal with stressful situations and maintain psychological balance. Nursing managers are encouraged to undertake courses on negative emotion management and interpersonal communication therapy in continuing education and learning, to improve the ability of nurses to deal with negative events and adopt positive coping methods, thereby reducing the level of psychological distress.
4. In the future, we will further examine the relationship between variables such as sleep quality, coping styles, and psychological distress in psychiatric nurses and analyze their mutual influence and the mechanism underlying their regulation. Through Balint group, group therapy, standardized interpersonal communication, and MBSR therapy, nurses can face negative events in life with a more positive attitude, focus on the problem, reduce bad mood, and improve the quality of their sleep, and ultimately improving the overall mental health.

We hope that through our findings, the Chinese government and authorities worldwide should pay attention to the mental health of nursing professionals, particularly in specialized hospitals. In addition, to lower the frequency of psychological distress, mental health education should be expanded globally to increase the quality and efficacy of psychological therapies, and ultimately establish a positive and pleasant work environment for nurses worldwide.

## Data availability statement

The raw data supporting the conclusions of this article will be made available by the authors, without undue reservation.

## Author contributions

JW was in charge of the data analysis and writing. ZZ was in charge of the data review and language modification. YT was in charge of the data collection and data entry. RZ was in charge of the data analysis and data entry. QL was in charge of the data collection and study design. BW and QS were in charge of essential help. All authors designed this study and contributed to and approved the final manuscript.

## Funding

This project was funded by Shandong Medical and Health Science and Technology Development Plan Project (2018WS296).

## Conflict of interest

The authors declare that the research was conducted in the absence of any commercial or financial relationships that could be construed as a potential conflict of interest.

## Publisher's note

All claims expressed in this article are solely those of the authors and do not necessarily represent those of their affiliated organizations, or those of the publisher, the editors and the reviewers. Any product that may be evaluated in this article, or claim that may be made by its manufacturer, is not guaranteed or endorsed by the publisher.

## References

[1] John-Henderson NA Williams SE Brindle RC Ginty AT. Changes in sleep quality and levels of psychological distress during the adaptation to university: The role of childhood adversity. Br J Psychol. (2018) 109:694–707. 10.1111/bjop.12314 29799113

[2] Feng D Ji L Xu L. Mediating effect of social support on the association between functional disability and psychological distress in older adults in rural China: does age make a difference? PLoS ONE. (2014) 9:e100945. 10.1371/journal.pone.0100945 24963867 PMC4070995

[3] Skillgate E Isacson Hjortzberg M Strömwall P Hallqvist J Onell C Holm LW. Non-Preferred work and the incidence of spinal pain and psychological distress-a prospective cohort study. Int J Environ Res Public Health. (2021) 18:10051. 10.3390/ijerph181910051 34639355 PMC8508031

[4] Weinberg A Creed F. Stress and psychiatric disorder in healthcare professionals and hospital staff. Lancet. (2000) 355:533–37. 10.1016/S0140-6736(99)07366-3 10683003

[5] Zou G Shen X Tian X Liu C Li G Kong L. Correlates of psychological distress, burnout, and resilience among Chinese female nurses. Ind Health. (2016) 54:389–95. 10.2486/indhealth.2015-0103 27021058 PMC5054279

[6] Lin HS Probst JC Hsu YC. Depression among female psychiatric nurses in southern Taiwan: main and moderating effects of job stress, coping behaviour and social support. J Clin Nurs. (2010) 19:2342–54. 10.1111/j.1365-2702.2010.03216.x 20659207

[7] Lu Q Zhong H. lnvestigation on the sleep quality of nurses of a certain mental hospital at grade lll level and analysis of the influencing factors. J Qilu Nurs. (2014) 21:10–2.

[8] Burnette JL Knouse LE Vavra DT O'Boyle E Brooks MA. Growth mindsets and psychological distress: a meta-analysis. Clin Psychol Rev. (2020) 77:101816. 10.1016/j.cpr.2020.101816 32163802

[9] Maridal HK Bjørgaas HM Hagen K Jonsbu E Mahat P Malakar S. Psychological distress among caregivers of children with neurodevelopmental disorders in Nepal. Int J Environ Res Public Health. (2021) 18:2460. 10.3390/ijerph18052460 33801567 PMC7967590

[10] Erdogan Yüce G Döner A Muz G. Psychological distress and its association with unmet needs and symptom burden in outpatient cancer patients: a cross-sectional study. Semin Oncol Nurs. (2021) 37:151214. 10.1016/j.soncn.2021.151214 34483014

[11] Sakamoto N Takiguchi S Komatsu H Okuyama T Nakaguchi T Kubota Y. Supportive care needs and psychological distress and/or quality of life in ambulatory advanced colorectal cancer patients receiving chemotherapy: a cross-sectional study. Jpn J Clin Oncol. (2017) 47:1157–61. 10.1093/jjco/hyx152 29077931

[12] Berg SS Rosenau PS Prichard JR. Sleep quality mediates the relationship between traumatic events, psychological distress, and suicidality in college undergraduates. J Am Coll Health. (2020) 1-4. 10.1080/07448481.2020.1826493 33073731

[13] Cairns KE Yap MB Pilkington PD Jorm AF. Risk and protective factors for depression that adolescents can modify: a systematic review and meta-analysis of longitudinal studies. J Affect Disord. (2014) 169:61–75. 10.1016/j.jad.2014.08.006 25154536

[14] Zhao X Li J Huang Y Jin Q Ma H Wang Y. Genetic variation of FYN contributes to the molecular mechanisms of coping styles in healthy Chinese-Han participants. Psychiatr Genet. (2013) 23:214–6. 10.1097/YPG.0b013e328364365d 23851594

[15] Wang Y Xiao H Zhang X Wang L. The role of active coping in the relationship between learning burnout and sleep quality among college students in China. Front Psychol. (2020) 11:647. 10.3389/fpsyg.2020.00647 32425843 PMC7204605

[16] Lin J Su Y Lv X Liu Q Wang G Wei J. Perceived stressfulness mediates the effects of subjective social support and negative coping style on suicide risk in Chinese patients with major depressive disorder. J Affect Disord. (2020) 265:32–8. 10.1016/j.jad.2020.01.026 31959583

[17] Roesch SC Adams L Hines A Palmores A Vyas P Tran C. Coping with prostate cancer: a meta-analytic review. J Behav Med. (2005) 28:281–93. 10.1007/s10865-005-4664-z 16015462

[18] Hoyt MA Thomas KS Epstein DR Dirksen SR. Coping style and sleep quality in men with cancer. Ann Behav Med. (2009) 37:88–93. 10.1007/s12160-009-9079-6 19194771

[19] Urfer-Maurer N Weidmann R Brand S Holsboer-Trachsler E Grob A Weber P. The association of mothers' and fathers' insomnia symptoms with school-aged children's sleep assessed by parent report and in-home sleep-electroencephalography. Sleep Med. (2017) 38:64–70. 10.1016/j.sleep.2017.07.010 29031758

[20] K Pavlova M Latreille V. Sleep disorders. Am J Med. (2019) 132:292–9. 10.1016/j.amjmed.2018.09.021 30292731

[21] Li Y Cong X Chen S Li Y. Relationships of coping styles and psychological distress among patients with insomnia disorder. BMC Psychiatry. (2021) 21:255. 10.1186/s12888-021-03254-7 34001068 PMC8130448

[22] An FR Qi YK Zeng JY Ding YM Chiu HF Ungvari GS. The prevalence of insomnia, its demographic correlates, and treatment in nurses working in Chinese psychiatric and general hospitals. Perspect Psychiatr Care. (2016) 52:88–94. 10.1111/ppc.12103 25639858

[23] Kalmbach DA Pillai V Arnedt JT Drake CL. DSM-5 Insomnia and short sleep: comorbidity landscape and racial disparities. Sleep. (2016) 39:2101–11. 10.5665/sleep.6306 27634805 PMC5103798

[24] Biddle DJ Hermens DF Lallukka T Aji M Glozier N. Insomnia symptoms and short sleep duration predict trajectory of mental health symptoms. Sleep Med. (2019) 54:53–61. 10.1016/j.sleep.2018.10.008 30529778

[25] Jamieson D Beaudequin DA McLoughlin LT Parker MJ Lagopoulos J Hermens DF. Associations between sleep quality and psychological distress in early adolescence. J Child Adolesc Ment Health. (2020) 32:77–86. 10.2989/17280583.2020.1811288 33206591

[26] Gómez-García T Ruzafa-Martínez M Fuentelsaz-Gallego C Madrid JA Rol MA Martínez-Madrid MJ. Nurses' sleep quality, work environment and quality of care in the Spanish National Health System: observational study among different shifts. BMJ Open. (2016) 6:e012073. 10.1136/bmjopen-2016-012073 27496241 PMC4985858

[27] Boivin DB Boudreau P. Impacts of shift work on sleep and circadian rhythms. Pathol Biol (Paris). (2014) 62:292–301. 10.1016/j.patbio.2014.08.001 25246026

[28] Zhang C Xiao S Lin H Shi L Zheng X Xue Y. The association between sleep quality and psychological distress among older Chinese adults: a moderated mediation model. BMC Geriatr. (2022) 22:35. 10.1186/s12877-021-02711-y 35012479 PMC8744230

[29] Gamze R Cantürk Y Cmert IT Ura S Yükselolu EH. Relationship between rem sleep behavior disorder and depression and anxiety and night eating syndrome. Prog Nutr. (2020) 22:e2021047. 10.23751/pn.v23i2.10130

[30] Fu W Wang C Zou L Guo Y Lu Z Yan S. Psychological health, sleep quality, and coping styles to stress facing the COVID-19 in Wuhan, China. Transl Psychiatry. (2020) 10:225. 10.1038/s41398-020-00913-3 32647160 PMC7347261

[31] Xiong W Liu H Gong P Wang Q Ren Z He M. Relationships of coping styles and sleep quality with anxiety symptoms among Chinese adolescents: a cross-sectional study. J Affect Disord. (2019) 257:108–15. 10.1016/j.jad.2019.07.032 31301610

[32] Tada A. The associations among psychological distress, coping style, and health habits in Japanese nursing students: a cross-sectional study. Int J Environ Res Public Health. (2017) 14:1434. 10.3390/ijerph14111434 29165395 PMC5708073

[33] Kashdan TB Barrios V Forsyth JP Steger MF. Experiential avoidance as a generalized psychological vulnerability: comparisons with coping and emotion regulation strategies. Behav Res Ther. (2006) 44:1301–20. 10.1016/j.brat.2005.10.003 16321362

[34] Kessler RC Andrews G Colpe LJ Hiripi E Mroczek DK Normand SL. Short screening scales to monitor population prevalences and trends in non-specific psychological distress. Psychol Med. (2002) 32:959–76. 10.1017/S0033291702006074 12214795

[35] Zhou C Chu J Wang T Peng Q He J Zheng W. Reliability and validity of 10-item Kessler Scale (K10) Chinese version in evaluation of mental health status of Chinese population. Chin J Clin Psychol. (2008) 16:627–9. 10.16128/j.cnki.1005-3611.2008.06.026

[36] Xie Y. A preliminary study on the reliability and validity of the simplified coping style scale. Chin J Clin Psychol. (1998) 6:114–5. 10.16128/j.cnki.1005-3611.1998.02.018

[37] Buysse DJ Reynolds CF. 3rd Monk TH Berman SR Kupfer DJ. The Pittsburgh Sleep Quality Index: a new instrument for psychiatric practice and research. Psychiatry Res. (1989) 28:193–213. 10.1016/0165-1781(89)90047-4 2748771

[38] Liu X Tang M Hu L Wang A Wu H Zhao G. Reliability and validity of the Pittsburgh sleep quality index. Chin J Psychiatry. (1996) 29:103–7.34534069

[39] Liu Y Yang C Zou G. Self-esteem, job insecurity, and psychological distress among Chinese nurses. BMC Nurs. (2021) 20:141. 10.1186/s12912-021-00665-5 34376216 PMC8353746

[40] Li T Zhang X Chen M Wang R He L Xue B. Psychological distress and its associated risk factors among university students. Rev Assoc Med Bras. (1992). (2020) 66:414–8. 10.1590/1806-9282.66.4.414 32578772

[41] Dong H Zhang Q Sun Z Sang F Xu Y. Sleep disturbances among Chinese clinical nurses in general hospitals and its influencing factors. BMC Psychiatry. (2017) 17:241. 10.1186/s12888-017-1402-3 28673267 PMC5496307

[42] Tsuboi H Tatsumi A Yamamoto K Kobayashi F Shimoi K Kinae N. Possible connections among job stress, depressive symptoms, lipid modulation and antioxidants. J Affect Disord. (2006) 91:63–70. 10.1016/j.jad.2005.12.010 16430969

[43] Torquati L Mielke GI Brown WJ Burton NW Kolbe-Alexander TL. Shift work and poor mental health: a meta-analysis of longitudinal studies. Am J Public Health. (2019) 109:e13–20. 10.2105/AJPH.2019.305278 31536404 PMC6775929

[44] Eanes L CE. The potential effects of sleep loss on a nurse's health. Am J Nurs. (2015) 115:34–42. 10.1097/01.NAJ.0000463025.42388.10 25793430

[45] Holen S Lervåg A Waaktaar T Ystgaard M. Exploring the associations between coping patterns for everyday stressors and mental health in young schoolchildren. J Sch Psychol. (2012) 50:167–93. 10.1016/j.jsp.2011.10.006 22386119

[46] Zhang RX Liu L Shi N Kong Y Li Y. The investigation and analysis of the stress coping of the patients with chronic insomnia and the enlightenment to the intervention. Med Philosophy. (2014) 35:88–90. 10.3969/j.issn.1002-0772.2014.16.034

[47] Wong HY Mo HY Potenza MN Chan MNM Lau WM Chui TK. Relationships between severity of internet gaming disorder, severity of problematic social media use, sleep quality and psychological distress. Int J Environ Res Public Health. (2020) 17:1879. 10.3390/ijerph17061879 32183188 PMC7143464

[48] Kain ZN Caldwell-Andrews AA. Sleeping characteristics of adults undergoing outpatient elective surgery: a cohort study. J Clin Anesth. (2003) 15:505–9. 10.1016/j.jclinane.2003.02.002 14698361

[49] Roberts RE Shema SJ Kaplan GA Strawbridge WJ. Sleep complaints and depression in an aging cohort: A prospective perspective. Am J Psychiatry. (2000) 157:81–8. 10.1176/ajp.157.1.81 10618017

[50] Alvaro PK Roberts RM. Harris JK Bruni O. The direction of the relationship between symptoms of insomnia and psychiatric disorders in adolescents. J Affect Disord. (2017) 207:167–74. 10.1016/j.jad.2016.08.032 27723540

[51] Teoh AN Kaur S Mohd Shukri NH Shafie SR Ahmad Bustami N Takahashi M. Psychological state during pregnancy is associated with sleep quality: preliminary findings from MY-CARE cohort study. Chronobiol Int. (2021) 38:959–70. 10.1080/07420528.2021.1902338 33779445

[52] Jarcho MR Slavich GM Tylova-Stein H Wolkowitz OM Burke HM. Dysregulated diurnal cortisol pattern is associated with glucocorticoid resistance in women with major depressive disorder. Biol Psychol. (2013) 93:150–8. 10.1016/j.biopsycho.2013.01.018 23410758 PMC3687535

[53] Alimoradi Z Broström A Tsang HWH Griffiths MD Haghayegh S Ohayon MM. Sleep problems during COVID-19 pandemic and its' association to psychological distress: a systematic review and meta-analysis. EClinicalMedicine. (2021) 36:100916. 10.1016/j.eclinm.2021.100916 34131640 PMC8192091

[54] Daut RA Fonken LK. Circadian regulation of depression: a role for serotonin. Front Neuroendocrinol. (2019) 54:100746. 10.1016/j.yfrne.2019.04.003 31002895 PMC9826732

[55] Golder SA Macy MW. Diurnal and seasonal mood vary with work, sleep, and daylength across diverse cultures. Science. (2011) 333:1878–81. 10.1126/science.1202775 21960633

[56] Dumont M Beaulieu C. Light exposure in the natural environment: relevance to mood and sleep disorders. Sleep Med. (2007) 8:557–65. 10.1016/j.sleep.2006.11.008 17383230

[57] Luan L Ren C Lau BW Yang J Pickard GE So KF. Y-like retinal ganglion cells innervate the dorsal raphe nucleus in the Mongolian gerbil (Meriones unguiculatus). PLoS ONE. (2011) 6:e18938. 10.1371/journal.pone.0018938 21552551 PMC3084235

[58] Zhou Z Wei L He Q Wang M Li D. Relationship between perceived stress and mental health among postgraduates: mediating role of sleep quality and moderating role of social support. Chin J Health Psychol. (2022) 30:749–52. 10.13342/j.cnki.cjhp.2022.05.022

[59] Sood A Sharma V Schroeder DR Gorman B. Stress Management and Resiliency Training (SMART) program among department of radiology faculty: a pilot randomized clinical trial. Explore (NY). (2014) 10:358–63. 10.1016/j.explore.2014.08.002 25443423

